# Prosthetic Aortic Valve Obstruction With Pannus Formation, an Approach for Diagnosis and Management: A Case Report

**DOI:** 10.7759/cureus.40416

**Published:** 2023-06-14

**Authors:** Aliaa Mousa, Hassaan Arshad, Tigran Kakhktsyan, Muhammad Humayoun Rashid, Jigar Patel, Harit Desai

**Affiliations:** 1 Internal Medicine, Capital Health Regional Medical Center, Trenton, USA; 2 Internal Medicine, Nishtar Medical University, Multan, PAK; 3 Cardiovascular Medicine, Capital Health Regional Medical Center, Trenton, USA

**Keywords:** mechanical thromb, fibro collagenous material, fluroscopy, aortic valve surgery, prosthetic valve pannus

## Abstract

Mechanical valve obstructions are critical medical and surgical emergencies that require immediate attention when patients present with new complaints and exhibit signs such as the onset of murmurs or the disappearance of mechanical valve clicks. Obstructions can arise from various causes, including pannus formation, thrombus, vegetations, or subvalvular tissue growth. While pannus formations have been previously reported for the mitral valve, they are less commonly observed in the aortic valve, and several hypotheses have been proposed to understand pannus formation. Accurate diagnosis relies on imaging techniques such as echocardiography and fluoroscopy, and surgical intervention is considered the optimal treatment approach. Here, we present the case of a 69-year-old female who had previously undergone aortic valve replacement and subsequently developed progressive dyspnea, fatigue, and a new onset murmur. Imaging modalities revealed a closed leaflet and a high transvalvular gradient over the valve. The patient underwent a prosthetic redo valve replacement, and post-surgery, she was discharged home without complications.

## Introduction

Mechanical prosthetic heart valves have revolutionized the management of valvular heart disease, providing life-saving treatment options for patients with severe valve dysfunction [1]. However, the long-term durability and function of these mechanical valves can be compromised by various complications [2], including mechanical valve obstruction. Among the causes of obstruction, pannus formation, characterized by the excessive growth of fibroelastic tissue around the mechanical prosthetic valve, has emerged as a significant concern.

Understanding the incidence, risk factors, and diagnostic approaches for mechanical prosthetic valve obstruction, particularly related to pannus formation, is vital for optimizing patient outcomes. In this study, we aimed to investigate the incidence of mechanical prosthetic valve thrombosis and evaluate the prevalence and clinical implications of pannus formation in patients with mechanical heart valves.

In this case, we are trying to shed light on the differential incidence and characteristics of mechanical prosthetic valve thrombosis and pannus formation. Additionally, we examined the diagnostic accuracy of various imaging modalities in identifying pannus overgrowth, as well as the optimal management strategies for patients presenting with mechanical prosthetic valve obstruction.

## Case presentation

A 69-year-old female with a past medical history of severe mitral regurgitation and aortic insufficiency status post complex mitral valve repair (P2 quadrangular resection, annular plication, 28 Physio-Ring, and Alfieri suture) with mechanical aortic valve replacement (#21 mm St Jude) in 2004 also has a history of polymyalgia rheumatica, iron deficiency anemia, insomnia, and hypothyroidism. Since 2004 she had been doing well and having regular follow-up visits with her cardiologist and cardiothoracic surgeon till 2023, she was on Warfarin with a targeted International Normalized Ratio (INR) of 2.5-3.5 which is being monitored weekly with a home machine and managed through her cardiologist. After 19 years, she started having gradual exertional dyspnea and requested to be brought in nonscheduled follow-up appointment with her cardiologist to discuss her current symptoms. On physical exam, auscultation revealed a systolic murmur: (3 out of 6 harsh holosystolic murmurs) and diastolic murmur: (Harsh 2 out of 6 blowing diastolic murmur at the aortic position), the patient underwent 2D echocardiography which did not visualize very well the mechanical aortic valve, which may have pannus formation which potentially responsible for the elevated velocities (PG/MG 129/80) and severe stenosis (MG 80) with normal function of her repaired mitral valve (MG 5), and mild-moderate tricuspid regurgitation. At that time given the concern for stenosis vs pannus formation, further evaluation in obtaining a transesophageal echo (TEE) was advised. TEE (Videos 1, 2) showed the limited opening of the mechanical prosthetic aortic valve with significant turbulence; however, the leaflets were not well seen due to acoustic shadowing. Her prior mitral valve repair remained intact with mild residual regurgitation, and she was noted to have mild-moderate tricuspid regurgitation.

**Video 1:** Transesophageal echocardiogram parasternal long axis showing aortic valve stenosis with moderate regurgitation.

**Video 2:** Transesophageal echocardiogram short axis showing severe aortic valve obstruction

Following the TEE she was taken for fluoroscopy (Valve Cine) of the aortic valve which revealed that one leaflet was stuck in the closed position and the other leaflet had restricted motion, there was an appreciation of mitral annuloplasty ring. That necessitated cardiovascular surgical evaluation, upon which hospital admission was recommended in order to bridge her anticoagulation status by starting intravenous heparin and discontinuing her current oral Warfarin dose. Thereafter, she underwent a redo sternotomy and redo mechanical aortic valve replacement (21 Inspiris). Intraoperatively, the mechanical prosthetic valve was found to be thrombosed. One leaflet was found to be completely closed and thrombosed. The other leaflet's motion was somewhat restricted. There was pannus formation as well as chronic thrombus. Post mechanical prosthetic valve implantation, a transesophageal echocardiogram showed normal biventricular function, mild tricuspid regurgitation, mild mitral regurgitation, and a trans-aortic valve gradient of 50. The patient was weaned successfully from the cardiopulmonary bypass machine and transferred to heart and vascular intensive care unit where she was weaned from mechanical ventilation and extubated. She was discharged from the hospital on the seventh-day post-operative after optimization and evaluated by physical and occupational therapy.

Later on, pathological examination of the excised tissue/prosthetic valve revealed fibro collagenous tissue with focal calcification in the pannus specimen, the second thrombus specimen showed blood clots and a resected explanted aortic prosthetic valve without vegetations.

## Discussion

Patients with mechanical valve replacement require lifelong close monitoring and follow-up with the primary cardiologist for monitoring for any new symptoms for which an immediate investigation should be undertaken [1]. Further, tight control of anticoagulation therapy is a must due to the potential for thrombogenicity [2]. Mechanical valve obstruction can be secondary to either pannus formation or thrombus formation or both. Multiple studies have found that mechanical prosthetic valve thrombosis is more common with the mitral valve compared to the aortic valve with an incidence that is five times higher for mitral valves [3]. According to a study conducted by Ellensen et al. [4], they found that the incidence of acute obstruction resulting from pannus formation was 0.7 per 1,000 cases with mechanical valve prostheses with a reported median time-to-mechanical prosthetic dysfunction of 11 years. This indicates that the development of pannus leading to valve obstruction is a relatively rare occurrence but can occur over an extended period following the initial implantation of the valve. Renzulli et al. [5] stated that fresh primary thrombosis is typically associated with recent perturbations in anticoagulation along with a recent onset of symptoms within 15 days, which can respond to fibrinolysis. On the other hand, progressive deterioration of clinical status is the usual presentation for pannus formation with a progressive increase in a gradient across the valve, and an abnormal hyperechogenic mass attached to the prosthesis [1]. Among the other reasons for mechanical dysfunction in an implanted aortic valve, the subvalvular tissues primarily consisting of the sinus of Valsalva, were found to be less likely to interfere significantly with the motion of the valve's disk(s) [6].

Pannus formation results from the extreme outgrowth of fibroelastic tissue secondary to non-immune reactions that start in the suture itself, then proliferate on the ventricular side of the mechanical valve. There are several risk factors associated with this finding, such as the technique of the operation, a rather small implanted mechanical prosthetic valve, ring, young age, female patients, prosthesis characteristics, low cardiac output with turbulent flow, infectious and inadequate anticoagulation [7].

Therefore, a hypothesis suggests that the occurrence of pannus overgrowth may be influenced by other factors. To investigate this, the expression of growth factors associated with neointimal thickening and inhibitory factors involved in extracellular matrix regulation was investigated, such as collagenase that was suggested by Schwartz et al. regarding coronary restenosis [8]. Neointimal hyperplasia is affected by numerous biochemical growth factors, including transforming growth factor (TGF-β) that has been identified for playing a role in extracellular matrix production, as well as smooth muscle cell migration and proliferation [7]. An upregulation of TGF-β expression may serve as a differentiation signal, prompting adventitial fibroblasts to transform into myofibroblasts, thereby contributing to arterial remodeling, similar to what is observed in coronary restenosis following percutaneous coronary intervention. Therefore, the presence of transforming growth factor (TGF-β) within the neointima of implanted devices or at the site of anastomosis in patients who have undergone procedures such as allogeneic vessel grafting, artificial vessel grafting, ventricular assist device placement, or coronary artery bypass grafting, can trigger the process of fibrogenesis with remodeling of the affected organ. It is conceivable that neointimal hyperplasia, driven by TGF-β expression, could also be involved in the development of pannus. Conversely, in this study [7], no expression of MMP-1, -3, or -9, which are known collagenases, gelatinases, and stromelysins, respectively, was observed. These factors were selected because of their in vitro substrate specificity and their inhibitory effect on neointimal development. Therefore, the involvement of matrix remodeling enzymes MMP-1, MMP-3, and MMP-9 in the formation of pannus tissue is improbable. Instead, the observed findings indicate that the excessive growth of pannus tissue is more likely attributed to the proliferation of myofibroblasts and the accumulation of extracellular matrix, which are stimulated by TGF-β. It is worth noting that MMPs have diverse functions and activities, and their roles in this context may differ.

The initial step for diagnosis of mechanical valve obstruction starts with a detailed history, followed by a physical examination, including cardiac auscultation with the patient in a forward/prone position, which most probably will demonstrate that the mechanical prosthetic clicks are muffled, or absent with the new onset of a murmur [9]. Thereafter, transthoracic echo (TTE) can be used to visualize the prosthetic valve and to quantify the severity of the mechanical prosthetic valve's function. But this can be limited by TTE due to acoustic shadowing [2,6] which was demonstrated in our patient. Transesophageal echo (TEE) is the next step that helps to differentiate the thrombus from the pannus (smaller and more echo-dense without mobility). Also, cardiac CAT scans [10] with a mass of high attenuation greater than the interventricular septum and HU of more than 145 units could be considered as a pannus, aiding in evaluating valvular and paravalvular pathologies. The manufacturer provides specific opening and closing angles for each type of prosthetic valve, which also vary depending on the valve's position, whether it's in the mitral or aortic. In CT scans, Bi-leaflet valves' normal opening angle ranges from 73° to 90° and 60° to 80° for mono-leaflet valves. However, fluoroscopy [9,10] is an essential imaging technique that provides a very reliable assessment of leaflet motion and valvular ring motion, as the range between a normal-opening angle to the closing angle ranges between less than 20 degrees to greater than 120 degrees-130 degrees. However, with a stuck leaflet, there is a limited range or absence of motion of the mechanical valve leaflet. Imaging techniques are not able to assess and differentiate the soft tissue associated with the valve and that is why surgery with pathological differentiation is the main diagnostic approach and treatment for pannus formation [1,10].

## Conclusions

The progressive malfunction of an implanted mechanical prosthetic valve may have catastrophic outcomes and necessitates a comprehensive approach. It requires elaborative detailed history taking and examination in symptomatic patients with mechanical prosthetic valves. Furthermore, TEE is crucial for differential diagnosis, as well as valve fluoroscopy. When making a treatment decision in these patients the degree of regurgitation or stenosis must be taken into consideration. Redo-Surgery with pathology referral is the gold standard approach for treatment.
